# The Evolution and Future Development of Attention Networks

**DOI:** 10.3390/jintelligence11060098

**Published:** 2023-05-23

**Authors:** Michael I Posner

**Affiliations:** Department of Psychology, Institute of Neuroscience, University of Oregon, Eugene, OR 97403, USA; mposner@uoregon.edu

**Keywords:** attention networks, alertness, orienting, executive, cognitive neuroscience, polymorphisms

## Abstract

The goal of this paper is to examine how the development of attention networks has left many important issues unsolved and to propose possible directions for solving them by combining human and animal studies. The paper starts with evidence from citation mapping that indicates attention has played a central role in integrating cognitive and neural studies into Cognitive Neuroscience. The integration of the fields depends in part upon similarities and differences in performance over a wide variety of animals. In the case of exogenous orienting of attention primates, rodents and humans are quite similar, but this is not so with executive control. In humans, attention networks continue to develop at different rates during infancy and childhood and into adulthood. From age four on, the Attention Network Test (ANT) allows measurement of individual differences in the alerting, orienting and executive networks. Overt and covert orienting do overlap in their anatomy, but there is evidence of some degree of functional independence at the cellular level. The attention networks frequently work together with sensory, memory and other networks. Integration of animal and human studies may be advanced by examining common genes involved in individual attention networks or their integration with other brain networks. Attention networks involve widely scattered computation nodes in different brain areas, both cortical and subcortical. Future studies need to attend to the white matter that connects them and the direction of information flow during task performance.

## 1. Introduction

The goal of this paper is to indicate similarities between many species in the orienting of attention and the differences between primates and humans in the executive network. Animal studies can be used to bring network and cellular studies together, partly because animal studies allow more invasive methods not used with humans. For example, cellular studies in primates have found different patterns of cellular activation for covert and overt orienting. In addition, by integrating pathways used to connect attention networks with memory, this paper attempts to clarify aspects of human and animal learning.

## 2. Centrality of Attention Networks

**Figure 1:**
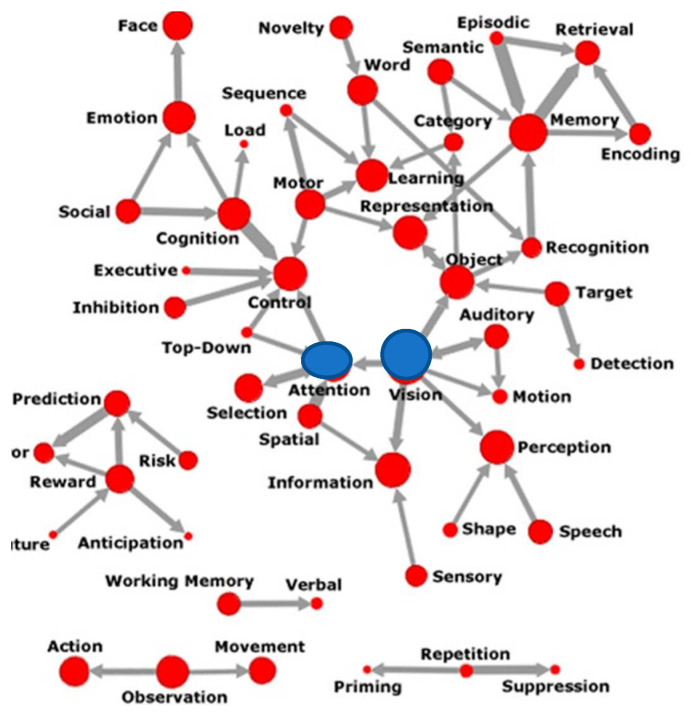
Semantics of Attention (Beam et al. 2014). Central nodes in blue. Permission to reprint from MIT press.

Recent citation analysis has traced the role of attention and vision to be at the center of the effort to create Cognitive Neuroscience as a field of study (Beam et al. 2014), as shown in Figure 1 below.

**Figure 2:**
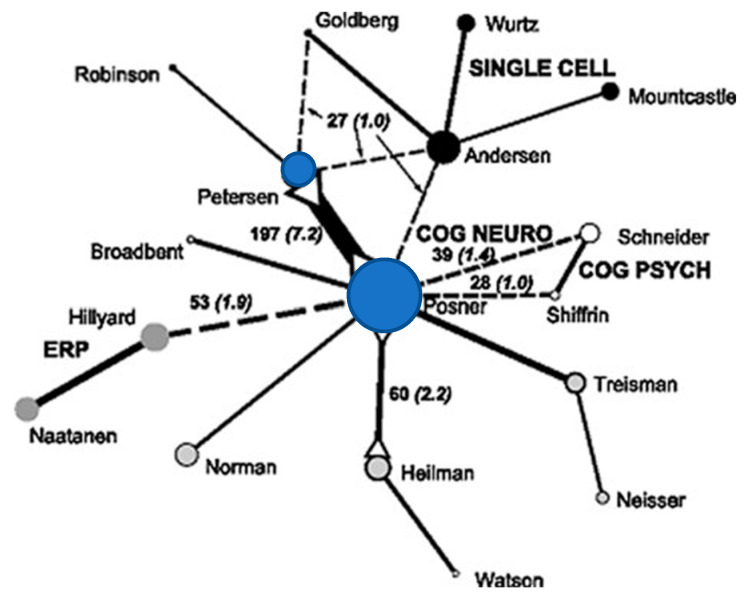
Connections among citations for the year 1995 between neurophysiology (black circles); cognition (open circles); ERP and Neurology (grey circles) showing Posner and Petersen (blue circles) as central connections. The numbers of co-citations are in black and those in ( ) are the % of all co-citations. Solid lines represent strong connections, dotted line weaker ones. The circle size represents the number of citations included in the analysis. Adapted with permission, Bruer (2010).

Another paper using a similar mapping method (see Figure 2) showed the connection between neurophysiological studies of attention (mostly cellular recording in primates (shown in black circles)) and cognitive studies (mostly reaction time (RT) and EEG recording, grey circles) (Bruer 2010). At the center of this work is our effort to bridge this divide using neurological patients, reaction time and imaging (blue circles and, for example, Posner and Petersen 1990).

## 3. Evolution of Attention

Attention did not appear on the scene with humans. It has a long evolutionary development within the animal kingdom. In this paper, we emphasize both similarities and differences in three attention networks between humans and other animals in the hope of illuminating some of the remaining problems in understanding how attention is implemented in the human brain and how it relates to other brain networks such as memory.

### 3.1. Animal Studies

One of the great advantages of using a variety of species for the same task is to gain perspective on the evolution of different aspects of attention. Table 1 examines orienting to a peripheral target that produces a faster RT when the subsequent target occurs at that location than when it occurs in the opposite visual field. Table 1 examines RT in that task in four species. Although these studies use somewhat varied conditions, the difference in RT between valid and invalid trials in all four species is strikingly similar.

The similarities across species shown in Table 1 are quite different from what is found in the executive attention network. The ability of non-human animals to resolve conflict is quite limited. For example, monkeys make about 25% errors after many weeks of training in a version of the Stroop effect developed for them. With less than one session of training, undergraduates make less than 3% errors on conflict trials (Washburn 1994). Even in the flanker task, which is easier than most Stroop versions, monkeys make about 20% errors with incongruent flankers even after considerable practice (Hassett and Hampton 2022). Humans seem to have much greater capacity to control their responses in the face of conflict than other primates, at least in this task.

### 3.2. Human Development

The ANT was developed to provide separate scores for three major networks involved: alerting, orienting and executive control (Fan et al. 2002). In one study of the development of attention networks of ages 6–9 years (Rueda et al. 2004a) using a child-friendly version of the ANT (Fan et al. 2002), there was a substantial reduction in the time to resolve conflict from 6 to 7 years of age. The four- and six-year olds shown in Table 2 made about 25% errors in the incongruent flanker condition, which is very similar to that of the monkeys discussed above, but by age seven and older errors were down to less than 5% and both RT and errors improved until adulthood. The orienting network showed relatively little improvement over this period. Thus, monkeys appear to resemble adult humans in exogenous orienting (Table 1), but are more like six-year olds in errors made in the flanker task. The table below indicates that human error rates are roughly stable between four and six, but decline remarkably by age seven and are stable up until nine.

Like RT, error rates are high for conflict trials in the early years and show a remarkable development between six and eight years of age. A possible reason for this developmental process arises in an fMRI study of the flanker effect from childhood through adulthood (Fjell et al. 2012). Up until age seven, the conflict score mainly correlates with the size of the right anterior cingulate cortex (ACC), while the overall improvement in RT continues to develop until adulthood and depends upon the degree of connectivity between the brain areas involved. Like reaction time, error rates are high for conflict trials in the early years and show a remarkable development.

### 3.3. Separability of Covert and Overt Attention

Much of attention involves overt changes, for example, in eye position and in motor preparation (Posner and Rothbart 1980). Certainly, measures of the time to move the eyes and to prepare a key press involve attention toward the intended targets. However, covert attention to a target can occur when eye position is held constant and the same motor response is made regardless of the target location. Of course, there are many cases where sensory and motor responses are irrelevant to the solution, for example in mathematical calculation or other forms of problem solving. It is clear that some forms of covert attention involve mechanisms not involved in movement. In the case of orienting the saccade system (overt attention) and covert orienting, there has been a debate over whether the same or different mechanisms are involved (Posner 1978; Rizzolatti et al. 1987).

The most intensely studied brain area that relates to the primacy of overt or covert attention in an evolutionary sense is the role of the frontal eye fields in shifts in attention. This interest goes back to a prefrontal motor theory of attention (Rizzolatti et al. 1987) in which the same mechanisms are involved in eye movements and covert orienting, but for covert orienting the eye movement is inhibited. Early imaging studies have clearly shown the highly overlapping brain areas involved in saccades and covert shifts in attention (Corbetta et al. 1998). It is clear that within the frontal eye fields, neurons activated during eye movements may be inhibited during covert shifts and those inhibited during eye movements may activated by purely visual events during covert shifts in attention (Gregoriou et al. 2012; Thompson et al. 2005). Recently, it has been reported using fMRI methods (Wu et al. 2022) that different but anticorrelated clusters of neurons predict either overt or covert attention. These findings show that overt and covert orienting are represented by differing functional clusters of neuronal populations in regions of the frontal eye fields and, thus, support the work with non-human primates.

It seems clear that even within the network involved in orienting, covert mechanisms are not identical to overt mechanisms at the cellular level. However, it may be reasonable to conclude, as Steinmetz and Moore (2012) do in their commentary on Gregoriou et al. 2012), “Perhaps it might be wise to consider that, at least within the FEF, all neurons participate in the control of covert and overt attention, but in separable ways” (p. 412). 

## 4. Interaction of Networks

The earliest ANT studies of the three attention networks appeared to show that they were independent in behavioral studies (Fan et al. 2002) and nearly so in imaging studies (Fan et al. 2005). However, as larger and more complex studies were designed, they provided evidence of interactions between the networks (Callejas et al. 2004, 2005; Fan et al. 2009). It would be unlikely that the networks could be completely independent since they must operate in situations where more than a single network needs to be involved. Thus, we can regard attention as a single system with at least three semi-independent networks performing largely different functions.

There have many studies describing how the orienting network interacts with different sensory modalities (Desimone and Duncan 1995; Posner 1978). It has been argued based on parietal lesion data that a single orienting network is the source of orienting, but the sites of its influence are found in different sensory modalities and brain areas (Posner 1988, Figure 8, p. 192). Subsequently, a distinction between a more dorsal voluntary and a more ventral exogenous orienting network has been elaborated on the basis of imaging data (Corbetta and Shulman 2002). These views contrast with the idea that each modality contains its own system for orienting.

**Figure 3:**
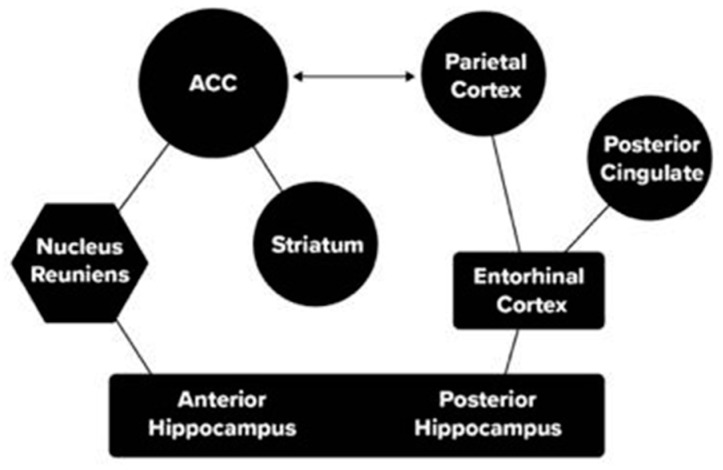
Pathways between attention nodes (circles) and memory nodes (rectangles) through the Nucleus Reuniens of the thalamus (hexagon) and entorhinal cortex. (Posner et al. 2022).

In addition, research has described the role of the parietal lobe and anterior cingulate (ACC) in the retrieval of memories (Weible 2013). In our studies of skill learning in mice, we used a task in which the mouse went in one direction (e.g., left) for a stimulus in the upper field and in the opposite direction for a stimulus in the lower field. We used optogenetics to suppress output from the ACC or Hippocampus with implanted lasers. We found that suppressing the ACC reduced the accuracy in the task at all stages of learning, supporting the critical role of the ACC in rodent recall (Weible et al. 2019). In general, the suppression of the ACC reduced performance overall more than was true of suppression of the hippocampus. Based on these findings and the literature, we have described two pathways through which attention and memory networks interact (See Figure 3).

The thalamic pathway has been identified with fear generalization to a new location in mouse studies of fear conditioning (Xu and Sudhof 2013), and in humans has been identified with storage of an unseen prototype of a set of figures which the person had learned to classify with the same response (Bowman and Zeithamova 2018). The pathway through the entorhinal cortex relates to human and monkey orienting. An extensive study of mice (Franco and Goard 2021) demonstrated the involvement of the retro splenial cortex (RSC) during the learning of a new skill that depends on memory for visual landmarks irrespective of the current location and direction of movement. The study found that, in addition to such spatial factors involved with head and eye position and movement trajectory, the RSC also incorporated memory for visuo-spatial landmarks irrespective of the direction of movement, which is an important context to long term memory for the environment. There was a posterior to anterior gradient on the role of context on performance, which was maximal in the posterior RSC population, weaker in the middle RSC and virtually absent in the anterior RSC throughout the trial.

Because the RSC cortex in mice involves interaction with the posterior cingulate and entorhinal cortex according to a summary of this anatomy (Alexander et al. 2022), its function clearly overlaps with the more posterior pathway connecting the parietal lobe to the hippocampus through the entorhinal cortex. This more posterior pathway is associated with spatial aspects of attention and appears dominant in navigation, which is a crucial aspect of rodent life. Moreover, the method of loci, one of the most prominent methods for improving human memory storage, may be effective in part because it makes use of this evolutionarily older pathway. The method developed in the Franco and Goard (2021) paper may allow more direct information on the role of neurons within this anatomy, and thus is the type of study that could allow progress in relating cellular physiology to the more general brain networks viewed by MRI. There is much yet to be learned concerning the function of each of the pathways shown in Figure 3, which we discuss below in the future studies section of this paper.

### Molecular Level

There is substantial evidence that relates a dominant neuromodulator with each of the attention networks studied by Beane and Marrocco (2004). There have been many questions raised about the replication of studies using the candidate gene approach (Zhu and Zhao 2007), which is the method used by the studies in Table 3. However, unlike many candidate gene studies, the genes here are associated with particular networks, and the studies use the ANT and other tasks to determine if a particular polymorphism is associated with the network that involves a specific neuromodulator. Findings have generally supported the association between genes and the network-related neuromodulators, as shown in Table 3. Another problem is that the candidate genes must have polymorphisms of sufficient frequency to allow testing with reasonably sized numbers. This means that all polymorphisms cannot be detected using the candidate gene method.

Table 3 specifies some genes that should be associated with one and only one network. These predictions have been tested and generally align with predictions, although there are exceptions in the literature.

Another approach to the molecular level deals with the interaction between attention and memory networks during the learning of skills (See Posner et al. 2022) and involves two pathways (illustrated in Figure 3). The mouse learning study discussed in the previous section allowed us to extract brain tissue from the ACC, HC and blood of mice before and after learning. We used a genome-wide association method to compare genes upregulated by learning in mouse brain and blood. We found three genes upregulated by learning in the mouse ACC and HC that were also upregulated in mouse blood. The use of blood allowed us to compare mouse blood with human blood extracted before and after two different forms of training. These were working memory and meditation training. One of the genes upregulated in mouse blood, CAP6 was also upregulated in human blood. All the significantly upregulated genes were related to a specific factor, NFkappaB, known to be involved in synaptic plasticity. While this study is preliminary, it does illustrate a possible method to bring together mouse and human learning at the molecular level.

## 5. Future Studies

It seems clear from this review that the study of attention has benefitted from relating the detailed behavioral properties of attention networks to the mechanisms that support them at the network, cellular and molecular levels. This is probably clearest within the orienting network, where the overall mechanisms of humans and animals are closely aligned. The finding that covert and overt exogenous orienting, while almost identical in behavior and in imaging, differ in the ways that individual neurons are used in the two types of orienting; this illustrates how many new things can be learned from using similar paradigms at the cellular and behavioral levels. However, much can still be learned from extending the work by comparing a larger array of animals. For example, one behavioral task, inhibition of return, has been reported in the archer fish (Gabay et al. 2013). Tracing performance in these tasks in invertebrates might further expand the evolutionary account.

The examination of how attention networks interact with other networks, as examined in Figure 3, is still very incomplete even in the case of memory. For one thing, we know relatively little about the direction of flow in subcortical areas where axons conducting information in different directions are within the same axonic bundle. We have proposed using a two virus method that may be able to solve this issue (Posner et al. 2022).

Tracing the evolution of attention networks requires more animals tested with similar behavioral tests. Comparing fMRI work in primates with cellular methods developed in rodents can help in these studies.

Much needs to be done to integrate human and animal studies of attention related to genes in order to further the framework suggested in Table 3. The use of the ANT (Fan et al. 2002) with primates and other non-human animals could help integrate these molecular studies.

## Figures and Tables

**Table 1:** Exogenous orienting of attention in four species.

	Valid	Invalid	Orienting (All in ms)
Humans *	380	410	30
Monkeys *	420	445	25
Rats *+	400	430	30
Mice +	420	450	30

Note: * RTs measured from Beane and Marrocco (2004); + RTs Measured from Wang and Krauzlis (2018) and Li et al. (2021).

**Table 2:** Error rates in a flanker task as a function of age.

Age	Congruent	Incongruent	Difference
4 *	12.4	21.9	9.5
6 +	8	23.6	15.6
7 +	5.4	5.9	0.5
8 +	4.9	4.7	−0.2
9 +	1.9	3.5	1.6

* from (Rueda et al. 2004b); + from (Rueda et al. 2004a).

**Table 3:** Relating attention networks to dominant modulators and selected relevant genes associated with the modulator. (Adapted from Posner et al. 2014).

Network	Modulator	Genes
Alerting	Norepinephrine	ADRA2A, NET
Orienting	Acetylcholine	CHRNA4, APOE
Executive	Dopamine	DRD4, DAT1, COMT, MAOA, DBH
	Serotonin	TPH2, 5HTT

## Data Availability

No new data is presented in this paper.
